# Exosome-related immune signatures and peripheral blood assays predict prognosis and immunotherapy response in hepatocellular carcinoma

**DOI:** 10.3389/fcell.2025.1696790

**Published:** 2026-01-26

**Authors:** Ruolian Gao, Lili Mi, Jianfei Shi, Meng Zhang, Xiaoling Duan, Man Zhao, Fei Yin

**Affiliations:** 1 Department of Gastroenterology, The Fourth Hospital of Hebei Medical University, Shijiazhuang, China; 2 Department of Pathology, The Fourth Hospital of Hebei Medical University, Shijiazhuang, China

**Keywords:** cancer-associated fibroblasts, exosome, fibroblast activation protein, hepatocellular carcinoma, immune therapy, tumor microenvironment

## Abstract

**Background:**

Exosomes are involved in cell-to-cell communication, and tumor-derived exosomes play an important role in the occurrence, development, and drug resistance of hepatocellular carcinoma (HCC) by regulating components in the tumor microenvironment (TME). However, the role of tumor-derived exosome-related immune (TDEI) genes in predicting HCC prognosis and immune therapy efficacy is not yet fully understood.

**Methods:**

The exoRBase2.0, GSE181946, and The Cancer Genome Atlas (TCGA) databases were used to analyze TDEI genes. Cox regression and least absolute shrinkage and selection operator (Lasso) analyses were used to identify TDEI genes that are closely related to the overall survival (OS) of patients with HCC. Subsequently, a predictive model was constructed based on the TCGA database and validated in the International Cancer Genome Consortium (ICGC) database. A nomogram was developed to predict survival. Immune infiltration analysis was used to estimate changes in immune cells and stromal cells in the TME. Immunohistochemistry (IHC), Western blot, and qRT-PCR demonstrated the regulation of TDEI genes on the TME. Peripheral blood assays were used for predicting immunotherapy response.

**Results:**

A combined prognostic model integrating S100A11 and PUSL1 expression with clinical characteristics was constructed, and it effectively predicted survival in HCC patients. S100A11 was associated with poorer immunotherapy efficacy. S100A11 regulated the TME by modulating interactions between cancer-associated fibroblasts (CAFs) and M2 macrophages. Experimental evidence demonstrated that S100A11 expression was associated with CAFs, M2 macrophage infiltration, and poorer progression-free survival (PFS). Interleukin-6 (IL-6), peripheral blood CD $8^{+}$ T cells, and prognostic nutritional index (PNI) can predict the response to immunotherapy.

**Conclusion:**

In this study, we identify a novel signature based on TDEI genes that has the potential to be a biomarker for predicting the prognosis and immunotherapy response for HCC. Peripheral blood tests can be used to predict the response to HCC immunotherapy. In our study, we provide an immunologic perspective for the development of precision therapy for HCC.

## 1 Introduction

Hepatocellular carcinoma (HCC) is one of the leading causes of cancer-related death worldwide (An et al., 2021). Patients with early-stage HCC can benefit from treatment options such as surgical resection, liver transplantation, and local therapies such as radiofrequency ablation. However, postoperative recurrence remains the main factor affecting the survival in these patients. For patients with advanced HCC, the combination of anti-vascular targeted drugs and immune checkpoint inhibitors (ICIs) has significantly improved treatment efficacy. However, nearly 30% of patients subsequently develop secondary drug resistance, which severely limits their survival benefits (Qin et al., 2023). Therefore, identifying high-risk patients with poor prognosis and those who are resistant to immunotherapy is a core clinical issue that urgently needs to be addressed.

Exosomes are bioactive, lipid bilayer nano-sized vesicles ranging in size from 30 to 150 nm. They contain RNA, proteins, and other bioactive molecules and can be secreted by virtually any cell (Tian et al., 2022). The tumor microenvironment (TME) is a complex structural mixture characterized by abnormal angiogenesis, chronic inflammation, and extracellular matrix remodeling, where it coexists and interacts with a variety of immune cells (Chen et al., 2023). In the TME of liver cancer, numerous cells contribute to tumor progression, and exosomes derived from both tumor cells and immune cells contribute to intercellular communication by transporting various substances, including proteins and RNA (Liu et al., 2023). Tumor-derived exosomes can be directly transported to CD $8^{+}$ T cells, natural killer (NK) cells, macrophages, and regulatory T cells, among others, thereby inhibiting their antitumor functions or modulating their cellular behavior (Zhang et al., 2021; 2020; Huang et al., 2022; Chen S.-W. et al., 2021; Zhao et al., 2020). Immune cells can also secrete exosomes. M2 macrophage-derived RNAs such as miR-23a-3p and miR-27a-3p promote liver cancer progression by promoting epithelial–mesenchymal transition (EMT) and angiogenesis, and by promoting HCC stemness (Lu et al., 2023; Li et al., 2021). In summary, exosomes play different roles by transmitting information between tumor cells and immune cells (Li et al., 2023).

Stromal cells are crucial components of the TME (Peng et al., 2022). The transformation of fibroblasts into cancer-associated fibroblasts (CAFs) is a core mechanism of immune evasion caused by altered TME, and exosomes play a crucial role in this process (Yin et al., 2019). Tumor cell-derived exosomal miR-1247-3p and RNPEP can contribute to the development and distant metastasis of HCC by promoting the transformation of fibroblasts into CAFs (Chen et al., 2025; Fang et al., 2018). CAFs interact with immune cells and tumor cells through exosomes and cytokines, which is a crucial pathway for altering the TME. CAF-derived exosomal circZFR promotes HCC progression and chemoresistance by inducing M2 macrophage polarization and enhancing cancer stemness (Zhou et al., 2022). In pancreatic cancer, CAF-derived exosomal NOD1 promotes pancreatic cancer development by regulating macrophage polarization toward M2 (Yang et al., 2025). CXCL9, CXCL10, and interleukin-6 (IL-6) secreted by CAFs promote HCC progression by inhibiting CD $8^{+}$ T-cell infiltration and promoting liver cancer cell proliferation (Gan et al., 2024; Zhao et al., 2019). In bladder cancer, CAF-secreted CXCL12 promotes immune resistance by promoting PD-L1 expression in tumor cells (Zhang et al., 2023). Exosomes play diverse roles by mediating communication among tumor cells, immune cells, and stromal cells, thereby remodeling the TME and promoting tumor initiation and progression, immune evasion, and resistance to immunotherapy (Wu et al., 2019).

In this study, we aimed to construct a predictive model for evaluating the prognosis and immunotherapeutic efficacy in patients with HCC and to explore the regulatory role of tumor-derived exosome-related immune (TDEI) genes in modulating the TME during HCC progression and the development of immunotherapy resistance. Our results demonstrated that TDEI genes may serve as prognostic biomarkers for HCC and contribute to immune resistance through remodeling of the TME. In addition, the IL-6 levels and peripheral blood CD $8^{+}$ T cells may serve as potential predictors of immunotherapeutic efficacy in patients with unresectable HCC.

## 2 Materials and methods

### 2.1 Data collection

Blood exosome transcriptomic data from HCC patients (n = 112) and healthy population (n = 118) were downloaded from exoRBase2.0 (http://www.exorbase.org/exoRBaseV2/toIndex). HCC transcriptomic data and corresponding clinical data were obtained from TCGA (n = 371) (https://portal.gdc.cancer.gov/projects/TCGA-LIHC/) and ICGC (n = 231) (https://dcc.icgc.org/projects/LIRI-JP) databases. In addition, transcriptome sequencing data for HCC before receiving anti-PD-1/PD-L1 therapy were obtained from the GEO database GSE181946 (https://www.ncbi.nlm.nih.gov/geo/).

### 2.2 Screening of TDEI genes

Differentially expressed genes in the exoRBase2.0 database were analyzed using the limma package in R software, and differentially expressed genes in TCGA and GSE181946 were analyzed using the DESeq2 package. In TCGA and exoRBase2.0, differentially expressed genes with corrected *p* < 0.05 and | log2 (fold change) |> 0.5 were selected for subsequent studies. In GSE181946, patients with efficacy of PR were included in the immune-responsive group and those with efficacy of PD were included in the immune-unresponsive group based on the assessment of anti-PD-1/PD-L1 treatment efficacy, and the differentially expressed genes with a corrected *p* < 0.05 and | log2 (fold change) |> 1 were selected for the follow-up study. Differentially expressed genes from the three databases were taken as intersections and defined as TDEI genes, and the VennDiagram package was used to draw the Wayne diagram.

### 2.3 Mutation analysis and copy number variation analysis

To analyze somatic mutation of TCGA-LIHC, “Masked Somatic Mutation” data were selected as the somatic mutation data of TCGA-LIHC through the TCGA website, and the R package maftools was used to visualize the somatic mutation situation. To analyze the copy number variation (CNV) in TCGA-LIHC, the “Masked Copy Number Segment” data were selected as the CNV data of TCGA-LIHC through the TCGA website. Then, the downloaded and processed CNV fragments were analyzed by GISTIC2.0.

### 2.4 Construction and validation of the prognostic model of risk score

Based on TCGA data, univariate Cox regression analysis was applied to screen out the TDEI genes affecting survival. Least absolute shrinkage and selection operator (Lasso) regression was performed using the “glmnet” package, and the optimal value of the penalty parameter (λ) was determined according to the 10-fold cross-validation used to select significant features, thereby excluding overfitting genes. Multivariate Cox regression analysis was then performed to derive the regression coefficients for the two prognostic TDEI genes (S100A11 and PUSL1), and the risk score was calculated as follows: risk score = sum (Expgene × coef). All HCC patients were categorized into high-risk and low-risk groups based on the risk score. The prognostic model’s predictive ability was assessed using time-dependent receiver operating characteristic (ROC) curves. The model was repeated using cancer data downloaded using ICGC (LIRI-JP) to test the accuracy of the predictive ability of the risk score.

### 2.5 Establishment of a nomograph model

Clinical characteristics and the risk score were analyzed by univariate and multivariate Cox regression analyses, and the prognostic model column chart was developed using the “Rms” package. The ROC curve was used to evaluate the differentiation of the model, the calibration curve was used to evaluate the calibration of the model, and the clinical decision curve was used to evaluate the clinical utility of the line-drawing model.

### 2.6 Immune infiltration analysis

For immune-cell infiltration analysis, the TIMER algorithm was used to estimate the infiltration levels of six major immune cell types in HCC samples. The CIBERSORT algorithm was applied to calculate the relative proportions of 22 immune cell subtypes for each HCC patient in the TCGA cohort. The EPIC algorithm was employed to determine the proportions of immune and stromal cells for each HCC patient in the TCGA cohort. In addition, the Tumor Immune Dysfunction and Exclusion (TIDE) algorithm was utilized to predict the potential immunotherapy response of HCC patients in the TCGA cohort based on the immunoscore.

The ESTIMATE algorithm was used to further evaluate the impact of immune- and stroma-related genes on patient prognosis. This algorithm infers the fraction of tumor and infiltrating normal cells in cancer tissues based on the gene expression profiles. The R package “estimate” was applied to the TCGA-LIHC expression data to calculate the immune, stromal, and estimate scores, thereby assessing the immune activity of the tumors.

### 2.7 Pathway enrichment analysis

Gene Ontology (GO) analysis is a common method for large-scale functional enrichment studies, including biological process (BP), cell component (CC), and molecular function (MF) terms. The Kyoto Encyclopedia of Genes and Genomes (KEGG) is a widely used database storing information on genomes, biological pathways, diseases, and drugs. The R package ClusterProfiler was used to perform GO and KEGG annotation analyses. The item screening criteria were *p* < 0.05 and FDR value (qvalue) < 0.05. The *p-*value correction method was the Benjamini–Hochberg procedure. To further evaluate phenotype-associated enrichment patterns, gene set enrichment analysis (GSEA) was conducted based on the predefined gene sets. Genes from the TCGA-LIHC cohort were ranked by their logFC values using the “clusterProfiler” package to identify gene sets showing significant coordinated expression changes associated with specific phenotypes.

### 2.8 Patients and clinical samples

The study cohort for Western blot, real-time quantitative PCR (qRT-PCR), and immunohistochemistry (IHC) was retrospectively selected from patients with HCC who underwent surgical resection at the Fourth Hospital of Hebei Medical University between January 2020 and December 2022. The inclusion criteria were as follows: age between 18 and 80 years, pathologically confirmed diagnosis of HCC, availability of postsurgical tumor tissue samples, and at least one measurable lesion according to the Response Evaluation Criteria in Solid Tumors (RECIST) version 1.1. The exclusion criteria included a history of systemic immunosuppressive therapy, the presence of other malignant tumors, and the presence of active autoimmune diseases requiring steroid treatment. Clinical information was collected from the hospital database and updated every 3 months through clinical visits or telephone follow-up.

The unresectable HCC cohort retrospectively enrolled 64 patients aged 18–80 years who received immune checkpoint inhibitors (ICIs) therapy at the Department of Gastroenterology, the Fourth Hospital of Hebei Medical University, between January 2023 and January 2025. HCC diagnosis was confirmed by pathological examination or laboratory and radiological evaluation according to the American Association for the Study of Liver Diseases guidelines, with the same exclusion criteria as above. Hematological parameters including the peripheral blood CD $8^{+}$ T-cell count, IL-6, complete blood count, albumin, alpha-fetoprotein (AFP), vitamin K deficiency or antagonist II-induced protein (PIVKA-II), and clinical characteristics were retrospectively collected within 7 days before the first ICIs treatment. The prognostic nutritional index (PNI) (Rimini et al., 2021) was derived from peripheral blood albumin (g/L) + 5 × absolute peripheral blood lymphocyte count ( $10^{9}$ /L). The efficacy of immunotherapy was assessed radiologically according to the modified Response Evaluation Criteria in Solid Tumors (mRECIST) criteria and categorized as complete response (CR), partial response (PR), stable disease (SD), or progressive disease (PD). According to the patients’ initial response to immunotherapy, they were categorized into the immunotherapy non-response group (PD) group or the immunotherapy response group, including CR/PR/SD.

### 2.9 IHC analysis

IHC was used to detect 103 HCC tumor tissues and five adjacent normal tissues from the Fourth Hospital of Hebei Medical University. These tissues were first fixed with formalin, then embedded with paraffin, and finally cut into 4- μ m-thick sections by a sectioning mechanism for later use. After antigen repair, the sections were incubated overnight at 4 °C with S100A11 (1:500 dilution, Proteintech), fibroblast activation protein (FAP) (1:300 dilution, Abways), or CD206 (1:2,000 dilution, Proteintech), and binding was detected using the avidin–biotin–peroxidase method. Slides were blocked after hematoxylin counterstaining. The slides were subsequently referred to two experienced pathologists for an independent, double-blind evaluation. For S100A11 and FAP, the staining intensity was scored on a scale of 0 (negative), 1 (weak), 2 (moderate), and 3 (strong). The staining extent was scored as 0 (0%), 1 (1%–25%), 2 (26%–50%), 3 (51%–75%), or 4 (76%–100%) according to the percentage of positively stained cells. The final IHC score was determined by multiplying the two scores, yielding a range from 0 to 12. For CD206-positive macrophages, the counting method included selecting five fields of view under a ×20 objective lens, counting the positive cells, and then taking the average value.

### 2.10 Western blot analysis

Three pairs of HCC tissues and paired adjacent normal tissues were used for Western blot. Proteins were extracted, quantified, and separated on 12% SDS–PAGE for S100A11 (1:7,500 dilution, Proteintech), 10% SDS–PAGE for FAP (1:2,000 dilution, Abways), and 6% SDS–PAGE for CD206 (1:1,500 dilution, Proteintech), followed by transfer onto PVDF membranes. Membranes were blocked with 5% nonfat milk blocking buffer, incubated with the primary antibodies at 4 °C overnight, and then incubated with an HRP-conjugated goat anti-rabbit secondary antibody (1:7,500, Boster Bio). Signals were visualized using an ECL detection kit (Boster Bio).

### 2.11 qRT-PCR analysis

Seven HCC tissue samples and seven HCC-adjacent normal tissue samples were used for qRT-PCR. Total RNA was extracted from human liver tissue using TRIzol reagent (Solarbio, China), and complementary DNA (cDNA) was synthesized by reverse transcription using 1 mg of RNA via the First-Strand cDNA Synthesis Kit. qRT-PCR assays were performed on the CFX96 Real-Time PCR Detection System (Bio-Rad, USA) utilizing SYBR Green-based fluorescence detection. The housekeeping gene β -actin was used as an internal control gene. Primer sequences for qPCR are provided in Supplementary Table S1.

### 2.12 Statistical analysis

For comparison between the two samples, the Student’s t-test was used for normally distributed data with homogeneous variance. For non-normally distributed data, the Wilcoxon test was used for paired data, and the Mann–Whitney test was used for unpaired data. The chi-square test was used for categorical data. A two-tailed *p* < 0.05 was considered statistically significant. R 4.4.1 was used for statistical analysis in this study.

### 2.13 Ethical approval

The study adhered to the guidelines outlined in the Helsinki Declaration and received approval from the Ethics Committee of the Fourth Hospital of Hebei Medical University (2025KS114). Given the retrospective design of the study, the committee waived the need for informed consent from all patients.

## 3 Results

### 3.1 Screening of TDEI genes

**FIGURE 1:**
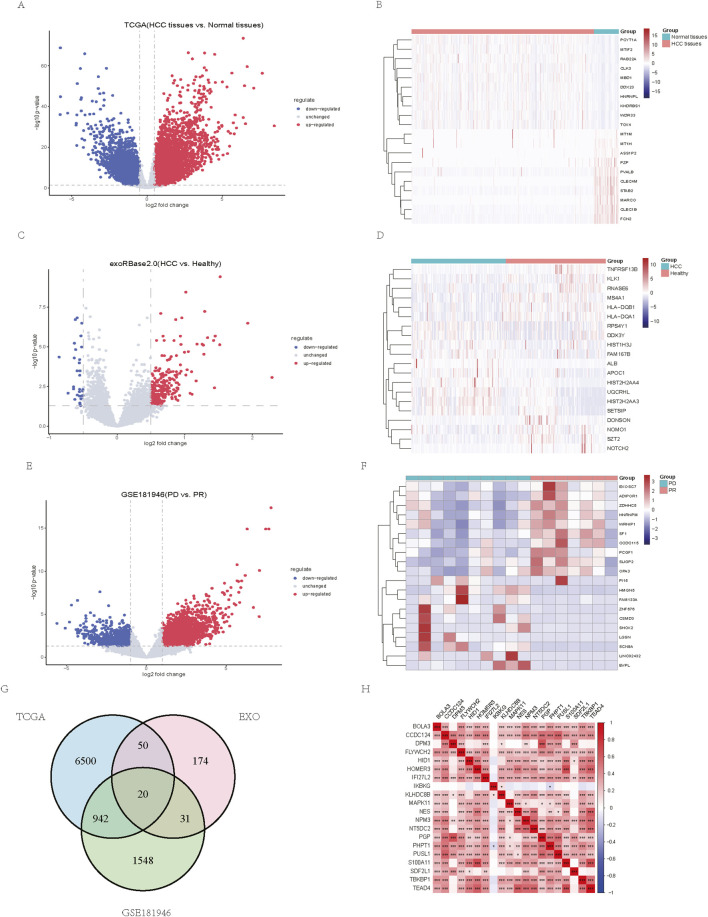
Identification of TDEI genes. **(A)** Volcano plot of differential gene expression between HCC tissues and normal tissues in TCGA-LIHC. **(B)** Top 20 differentially expressed genes between HCC tissues and normal tissues in TCGA-LIHC. **(C)** Volcano plot of differential gene expression between HCC patients and healthy population in exoRBase2.0. **(D)** Top 20 differentially expressed genes between HCC patients and healthy population in exoRBase2.0. **(E)** Volcano plot of differentially expressed genes between the PD and PR groups in the GSE181946 database. **(F)** Top 20 differentially expressed genes between the PD and PR groups in the GSE181946 database. **(G)** Intersection of differentially expressed genes in TCGA-LIHC, exoRBase2.0, and GSE181946. **(H)** Correlation heatmap of TDEI genes in TCGA-LIHC. *p < 0.05, **p < 0.01, and ***p < 0.001.

We compared differentially expressed genes between HCC tumor tissues and normal tissues in TCGA-LIHC and found 7,512 genes highly expressed in tumor tissues and 3,087 genes lowly expressed in tumor tissues (Figures 1A,B). We also compared exosome-related genes between HCC patients and the healthy population in exoRBase2.0, identifying 306 differentially expressed genes, of which 275 were highly expressed and 31 were lowly expressed in the HCC group (Figures 1C,D). Furthermore, we compared the differentially expressed genes between the immune-responsive group and the immune-unresponsive group in GSE181946 and found 2,541 highly expressed genes and 961 lowly expressed genes in the immune-unresponsive group (Figures 1E,F). A heatmap shows the top 20 differentially expressed genes. To investigate the role of TDEI genes as signature genes for prognosis and immunotherapy resistance, we selected genes that were highly expressed in both HCC tumor tissues and exosomes. By intersecting these differentially expressed genes with genes that were highly expressed in the immune-unresponsive group, we generated 20 TDEI genes (Figure 1G). A heatmap depicts the relationships between TDEI genes in TCGA-LIHC (Figure 1H).

### 3.2 Mutation analysis and copy number variation analysis

**FIGURE 2:**
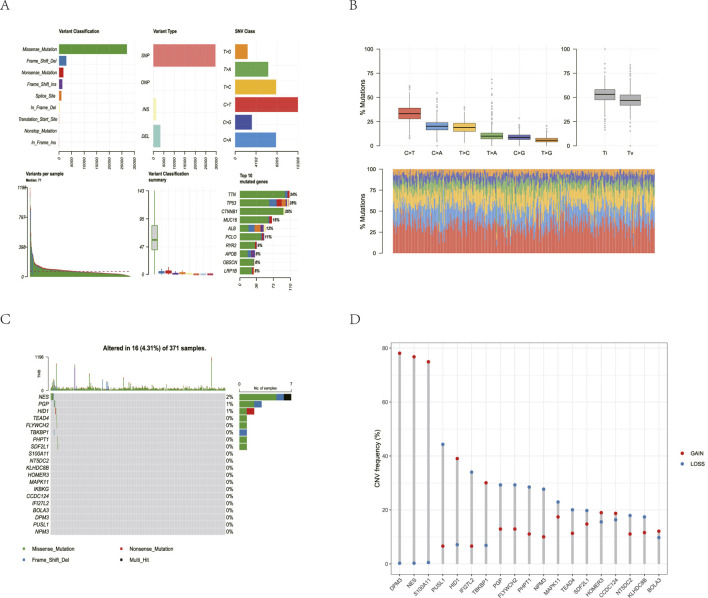
Mutation analysis of TDEI genes in TCGA-LIHC. **(A)** Mutation presentation in TCGA-LIHC. **(B)** Boxplot and stacked bar plot of TCGA-LIHC mutation types. **(C)** Mutation waterfall plot in TDEI gens in TCGA-LIHC. **(D)** CNV of TDEI gens in TCGA-LIHC.

To investigate the somatic mutation spectrum of 20 TDEI genes in TCGA-LIHC, mutational analysis was performed on HCC patient samples, and the results were compiled and analyzed. The results showed that missense mutations, frameshift deletions, nonsense mutations, and frameshift insertions were the predominant somatic mutations in TCGA-LIHC, with missense mutations being the most common. Furthermore, TDEI genes in HCC patients primarily manifested as single nucleotide polymorphisms (SNPs), along with some deletions (DELs) and insertions (INS). C > T was the most common single nucleotide variant (SNV), followed by T > C and C > A (Figures 2A,B). Somatic mutations were found in eight of the 20 TDEI genes, including NES and PGP. These genes were implicated in 16 of the 371 samples harboring somatic mutations, representing 4.31% of the total samples. Mutational status in TCGA-LIHC is presented in a bar chart (Figure 2C). GISTIC2.0 analysis was used to identify CNVs in TCGA-LIHC, and the results showed that 19 of the 20 TDEI genes exhibited CNVs (Figure 2D).

### 3.3 Constructing a risk prognosis model in TCGA-LIHC

**FIGURE 3:**
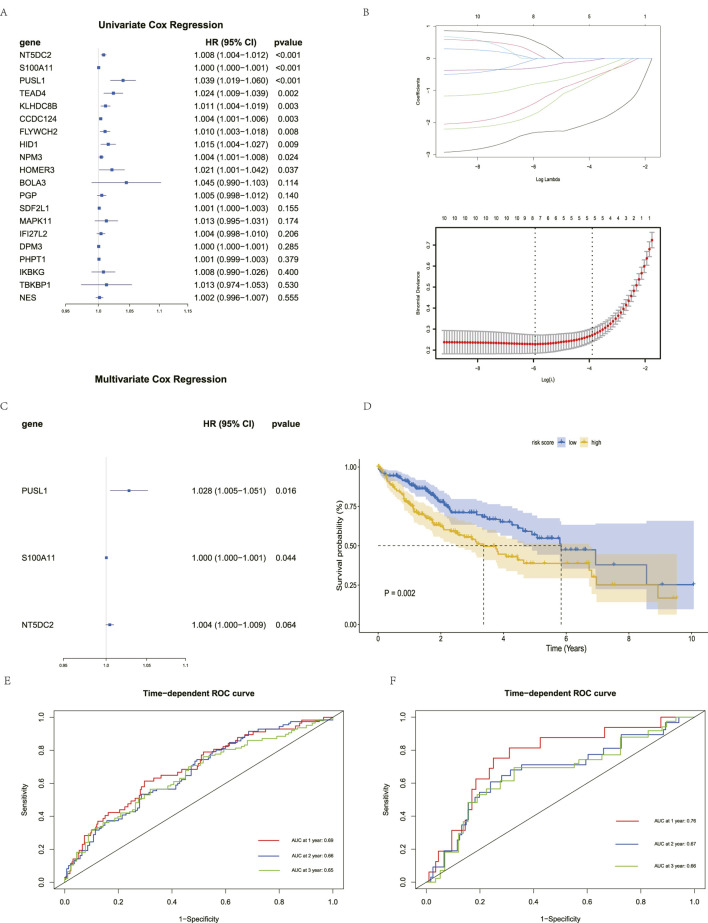
Construction of two prognostic TDEI genes. **(A)** Forest plot of univariate Cox regression analysis. **(B)** Lasso regression analysis of prognosis-related DEGs. **(C)** Forest plot of multivariate Cox bidirectional stepwise regression analysis. **(D)** Kaplan–Meier survival curves in TCGA-LIHC. **(E)** ROC analysis predicting the prognostic value of 1-, 2-, and 3-year OS rates in TCGA-LIHC. **(F)** ROC analysis predicting the prognostic value of 1-, 2-, and 3-year OS rates in LIRI-JP.

To further evaluate the prognostic ability of TDEI genes in HCC patients, we performed Cox regression and Lasso regression analyses based on TCGA-LIHC patient survival data. First, univariate Cox regression analysis revealed that 10 TDEI genes (S100A11, NT5DC2, KLHDC8B, TEAD4, HOMER3, FLYWCH2, CCDC124, HID1, PUSL1, and NPM3) were significantly associated with the overall survival (OS) in HCC patients (Figure 3A). Lasso regression was used to narrow the range, selecting the lambda value that minimized the mean variance of the model. A total of eight TDEI genes (S100A11, NT5DC2, KLHDC8B, TEAD4, FLYWCH2, CCDC124, HID1, and PUSL1) were identified (Figure 3B). Finally, multivariate Cox bidirectional stepwise regression analysis identified two prognostic TDEI genes significantly associated with OS in HCC patients: S100A11 and PUSL1 (Figure 3C). Based on the expression and regression coefficients of these two genes, we constructed a risk prognostic score according to the following formula: risk score = 0.00026 × S100A11 + 0.02752 × PUSL1. To explore the impact of the risk score on patient prognosis, we stratified HCC patients into high-risk and low-risk groups based on the median risk score. We found that the OS rate in the high-risk group was significantly lower than that in the low-risk group (Figure 3D). Using AUC, we found that the risk prognostic scores were accurately predictive in 1-, 2-, and 3-year survival assessments in TCGA-LIHC (1-year AUC = 0.69, 2-year AUC = 0.66, and 3-year AUC = 0.65) (Figure 3E). We validated the findings in the LIRI-JP cohort from ICGC, with similar results. The AUC values of the risk prognostic score for 1-year, 2-year, and 3-year survival evaluation were 0.76, 0.67, and 0.66, respectively, indicating that the risk model has wide applicability (Figure 3F).

### 3.4 Correlation between the two prognostic TDEI genes and clinical case characteristics

**FIGURE 4:**
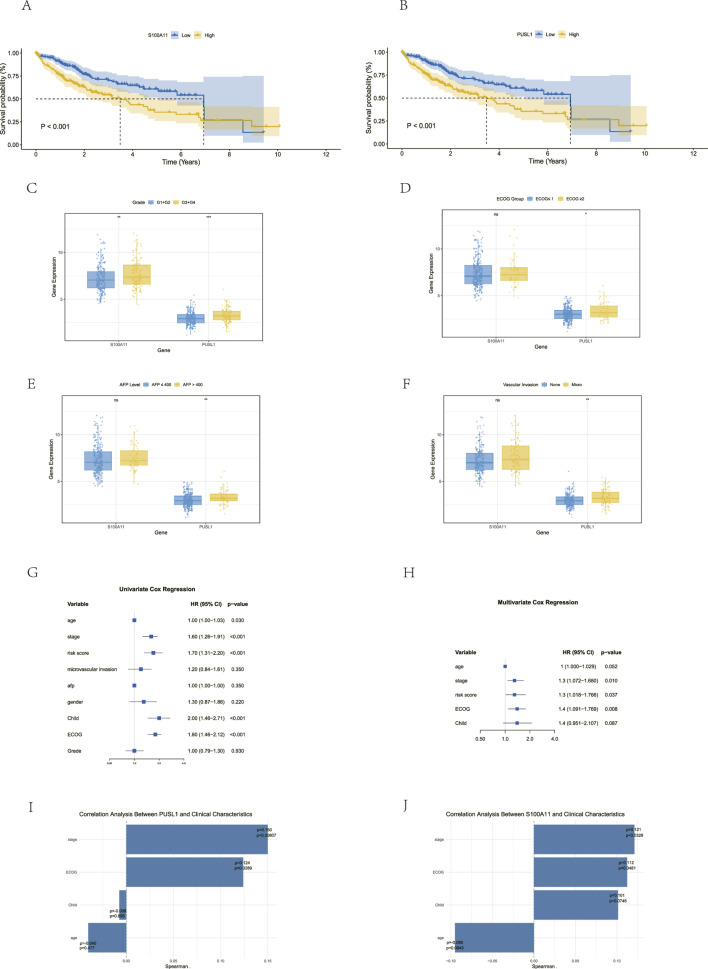
Study on the correlation between the clinical and pathological features. **(A)** Kaplan–Meier survival curves of S100A11 in TCGA-LIHC. **(B)** Kaplan–Meier survival curves of PUSL1 in TCGA-LIHC. **(C)** Correlation between gene expression and tumor grade (G1+G2 vs. G3+G4). **(D)** Correlation between gene expression and ECOG ( ≤ 1 vs. ≥ 2). **(E)** Correlation between gene expression and AFP level. **(F)** Correlation between gene expression and MVI status. **(G,H)** Univariate Cox regression analysis and multivariate Cox regression analysis of survival of HCC patients in TCGA-LIHC. **(I)** Correlation analysis of PUSL1 with clinical characteristics. **(J)** Correlation analysis of S100A11 with clinical characteristics.

We further validated the model by investigating the correlation between the expression levels of the two prognostic TDEI genes and various clinical characteristics. First, we analyzed the relationship between the expression levels of the two prognostic TDEI genes and survival. The results showed that high expression of S100A11 and PUSL1 was associated with worse overall survival (*p* < 0.05) (Figures 4A,B). Subsequently, we further analyzed the correlation between the two prognostic TDEI genes and clinico-pathological features of HCC. Regarding histopathological grade, high expression of S100A11 (*p* < 0.01) and PUSL1 (*p* < 0.001) was significantly associated with a worse HCC histological grade (Figure 4C). PUSL1 expression was significantly increased in patients with AFP > 400 U/mL, eastern cooperative oncology group (ECOG) performance status score ≥ 2, or microvascular invasion (MVI) (Figures 4D–F). The consistency between these two prognostic TDEI genes and clinical characteristics reinforced the reliability of the model.

### 3.5 Constructing a nomogram for the prediction model

**FIGURE 5:**
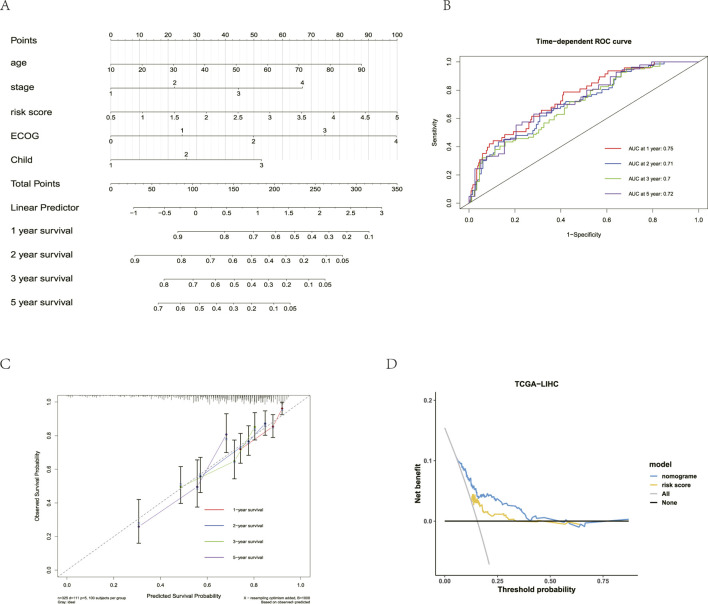
Construction and evaluation of nomograms. **(A)** Prognostic nomogram of multivariate Cox regression model. **(B)** ROC analysis predicts the prognostic value of 1-, 2-, 3-, and 5-year OS rates in TCGA-LIHC. **(C)** Prognostic calibration curve. **(D)** Prognostic calibration curve.

We constructed a nomogram based on the risk score and clinical and pathological characteristics to provide more accurate prognostic prediction. Univariate Cox regression analysis showed that the age, tumor stage, risk score, ECOG score, and Child–Pugh grade were factors influencing OS in HCC patients. Multivariate Cox regression analysis confirmed that the tumor stage, risk score, and ECOG score were independent prognostic factors (Figures 4G,H). We further analyzed the correlation among PUSL1, S100A11, and clinical characteristics and found that PUSL1 was positively correlated with the stage (ρ=0.150, p<0.05) and ECOG (ρ=0.124, p<0.05) and that S100A11 was positively correlated with the stage (ρ=0.121, p<0.05) and ECOG (ρ=0.112, p<0.05) (Figures 4I,J). Consistent with clinical practice and previous studies, the age and Child–Pugh grade also affected the prognosis in patients with HCC. In multivariate Cox regression analysis, the *p-*values for age and Child’s disease stage were 0.052 and 0.087, respectively, with both less than 0.1. Therefore, the age and Child–Pugh grade were also included in the prediction model. Subsequently, a nomogram incorporating the age, tumor stage, risk score, ECOG score, and Child–Pugh grade was constructed (Figure 5A). Using the nomogram, each patient was assigned a score for each predictor, and a total score was calculated by summing these scores. The 1-, 2-, 3-, and 5-year OS rates were then obtained by transforming the total score. In TCGA-LIHC, the ROC curve showed that the prediction model exhibited robust predictive ability (Figure 5B), with AUC values of 0.75 (1 year), 0.71 (2 years), 0.7 (3 years), and 0.72 (5 years). The calibration curve confirmed the reliability and predictive ability of the model (Figure 5C). Clinical decision curve analysis showed that the model exhibited the best clinical benefit when clinical characteristics and risk scores were combined to predict clinical outcomes, clarifying the clinical significance of the nomogram (Figure 5D). All these indicate good consistency between the predicted results and the observed results.

### 3.6 Immune-cell infiltration analysis

**FIGURE 6:**
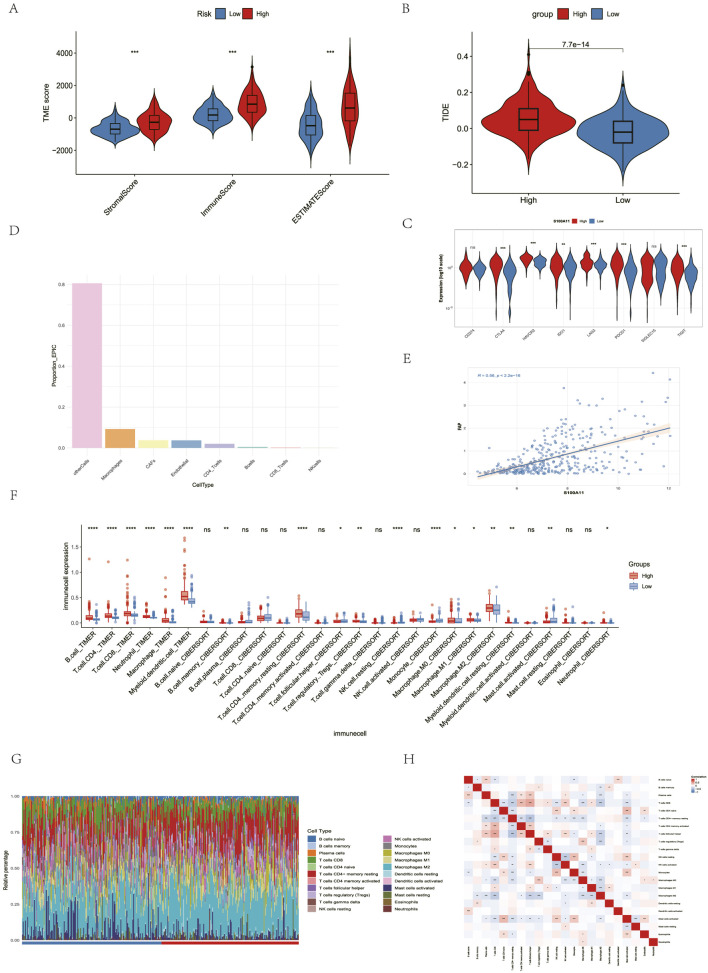
Immune infiltration analysis. **(A)** ESTIMATE analysis between high and low risk score groups. **(B)** TIDE scores between high and low risk score groups. **(C)** Distribution of immune checkpoints between high and low S100A11 groups. **(D)** EPIC analysis between high and low S100A11 groups. **(E)** Correlation analysis between S100A11 and FAP in TCGA-LIHC. **(F)** Comparison of immune cell infiltration abundance between high and low S100A11 groups. **(G)** Stacked bar chart of immune cell infiltration abundance between high and low S100A11 groups. **(H)** Heatmap of infiltration abundance correlation between immune cells. *p < 0.05, **p < 0.01, and ***p < 0.001.

In clinical practice, combined immunotherapy has significantly improved the prognosis of HCC patients. Immunotherapy exerts its antitumor effects by regulating the TME. Therefore, we continue to further investigate the relationship between TDEI genes and the TME. Current research suggests that S100A11 in liver disease is primarily associated with promoting liver fibrosis (Zhu et al., 2022), the development of nonalcoholic fatty liver disease Teng et al. (2021), and the occurrence and metastasis of liver cancer Zheng et al. (2023). Based on these studies, we focused on analyzing the role of S100A11 in regulating the TME. To investigate whether S100A11 affects the TME in HCC, we evaluated the relationship between S100A11 expression levels and TME composition. Overall, the stromal score, immune score, and estimate score were higher in the S100A11 high-expression group than in the low-expression group (Figure 6A). Furthermore, immune checkpoints CTLA-4, HAVCR-2, IDO1, LAG-3, PDCD-1, SIGLEC-15, and TIGIT were upregulated in the S100A11 high-expression group and downregulated in the S100A11 low-expression group (Figure 6C). These results suggest that the S100A11 high-expression group has an immune-hot phenotype. The TIDE score predicted the relationship between S100A11 expression level and immunotherapy efficacy, suggesting that the S100A11 high-expression group had a poorer immunotherapy response (Figure 6B). We further analyzed stromal and immune cells in the TME using the EPIC algorithm. HCC patients were divided into high-expression and low-expression groups based on S100A11 expression levels. The results showed that macrophages and CAFs were the two most abundant cell types among the eight cell types (Figure 6D). In TCGA-LIHC, the expression of S100A11 was positively correlated with that of FAP, a marker for CAFs (Figure 6E). Based on the pro-fibrotic effect of S100A11, we categorized CAFs into high-infiltration and low-infiltration groups based on the EPIC score. Using TIMER and CIBERSORT, we directly analyzed the relationship between immune cell types and CAFs in HCC tissues. The results revealed that in the high-infiltration CAF group, immunosuppressive cells such as Tregs and M2 macrophages were significantly infiltrated, while the infiltration of cytotoxic cells such as NK cells and monocytes was significantly reduced (Figures 6F–H). Among these immune cells, M2 macrophages were the most abundant, indicating that CAFs are associated with high M2 macrophage infiltration and that CAFs may promote the transformation of macrophages to the M2 phenotype. CAFs have also been shown to regulate the transformation of macrophages to the M2 phenotype in other cancer types. Therefore, our subsequent studies will explore the interaction between S100A11 and macrophages and CAFs.

### 3.7 Pathway enrichment analysis

**FIGURE 7:**
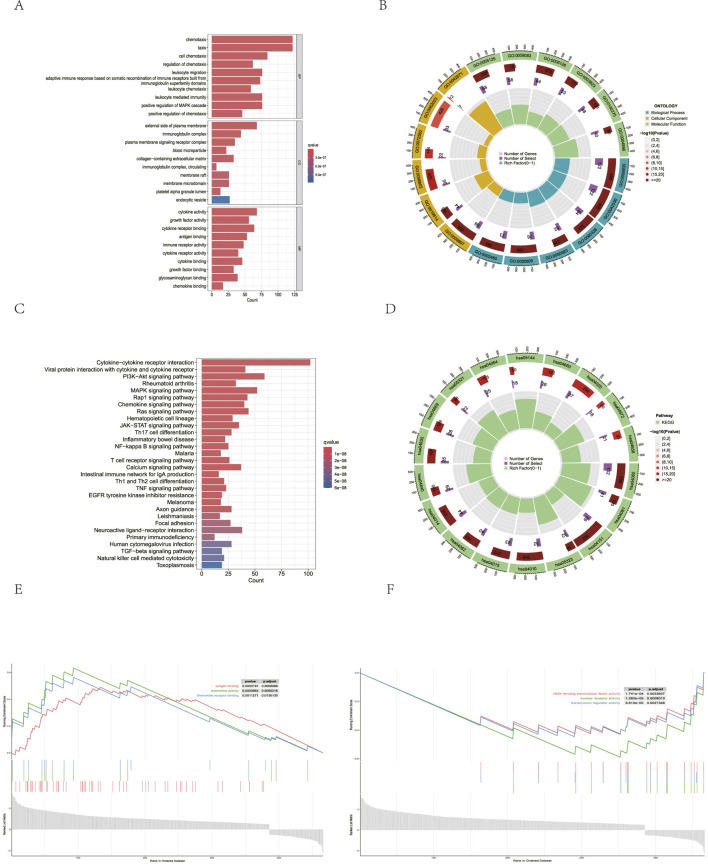
CAF-related pathway analysis. **(A,B)** Bar plot graph and circle plot of GO enrichment analysis. **(C,D)** Bar plot graph and circle plot of KEGG enrichment analysis. **(E)** GSEA of the high-infiltration CAF group. **(F)** GSEA of the low-infiltration CAF group.

To explore the biological behaviors and signaling pathways associated with CAFs, we performed pathway enrichment analysis. Using the EPIC algorithm, the TCGA-LIHC cohort was divided into a high-infiltration CAF group and a low-infiltration CAF group based on the degree of CAF infiltration. The limma package was used to analyze differentially expressed genes between the two groups. The intersection with immune-related genes in the ImmPort database yielded 466 genes, which were defined as CAF-related genes. GO and KEGG analyses revealed associations with biological behaviors and signaling pathways, including immune cell migration, cytokine activity, MAPK signaling, PI3K-AKT signaling, and TGF- β signaling (Figures 7A–D). Furthermore, the GSEA identified distinct molecular functions between the two groups. In the high-infiltration CAF group, immune-related functions such as cytokine activity were key, whereas in the low-infiltration CAF group, transcriptional regulation was the key function (using adjusted *p* < 0.05 and FDR < 0.25 as the screening criteria) (Figures 7E,F). According to relevant studies, the TGF- β signaling pathway is mainly involved in the activation of CAFs, which is consistent with our previous research results (Ringuette Goulet et al., 2018).

### 3.8 Evidence of S100A11 expression and regulation of the TME in HCC

**FIGURE 8:**
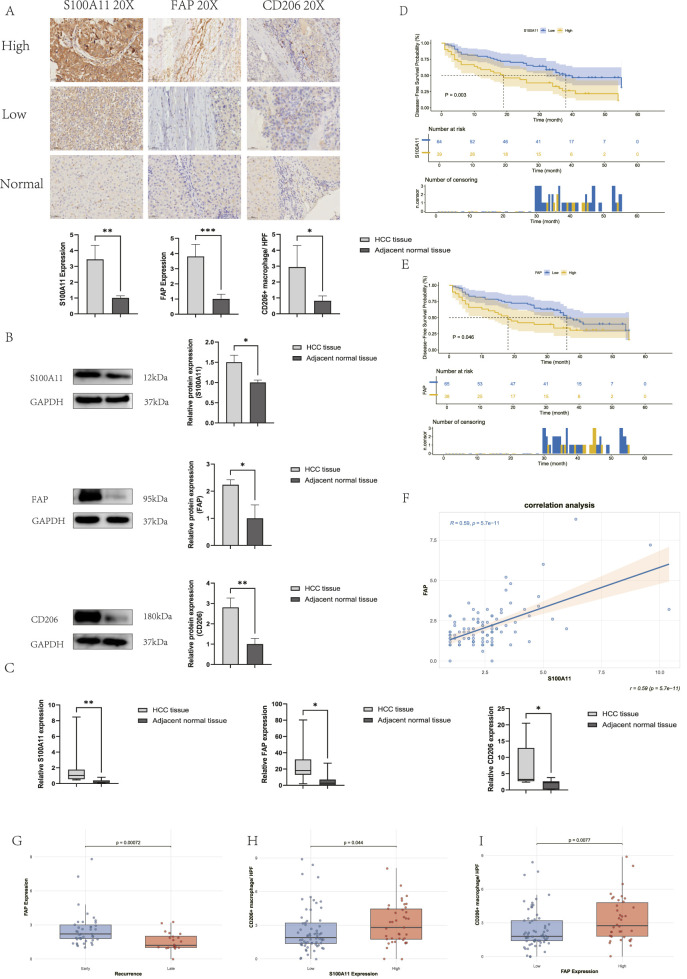
Expression and prognostic significance of S100A11, CAFs, and M2 macrophages. **(A)** Expression difference in HCC tissues and adjacent normal tissues in IHC, where the tumor tissues include those with both high expression and low expression. **(B)** S100A11, FAP, and CD206 expression in tumor and adjacent normal tissues in Western blot. **(C)** Expression difference in the tumor and adjacent normal tissues in qRT-PCR. **(D)** Comparison of PFS between high and low IHC score groups of S100A11 in HCC tissues. **(E)** Comparison of PFS between high and low IHC score groups of FAP in HCC tissues. **(F)** Correlation analysis between S100A11 and FAP in IHC score of HCC. **(G)** Differences between early and late recurrence in FAP. **(H)** Differences in CD206+ macrophages between S100A11 high-expression and low-expression groups. **(I)** Differences in CD206+ macrophages between FAP high-expression and low-expression groups.

To investigate the relationship between S100A11 and CAFs and M2 macrophages and its prognostic impact in HCC patients, we performed IHC analysis. FAP, a marker for activated CAFs, and CD206, a marker for M2 macrophages, were used to assess the expression levels of S100A11, FAP, and CD206 by IHC and conduct correlation analysis. First, IHC revealed that the expression levels of S100A11, FAP, and CD206 were higher in tumor tissues than in adjacent normal tissues (Figure 8A). Consistently, Western blot analysis further confirmed the elevated expression of S100A11, FAP, and CD206 in tumor tissues compared with that in matched adjacent normal tissues (Figure 8B). Moreover, qRT-PCR analysis verified that the mRNA levels of these genes were also significantly elevated in tumor tissues (Figure 8C). Second, we analyzed the relationship between S100A11 and FAP expression and progression-free survival (PFS). The median PFS was significantly lower in the S100A11 high-expression group than in the low-expression group (19 months vs. 38.5 months) (Figures 8A,D), and the median PFS was significantly lower in the FAP high-expression group than in the low-expression group (18 months vs. 36 months) (Figures 8A,E). We also found a significant positive correlation between S100A11 expression and FAP expression (Figure 8D). Similar results were observed in the TCGA-LIHC cohort (Figure 6E). Furthermore, CD206+ macrophage counts were significantly elevated in both the S100A11 and FAP high-expressing groups (Figures 8H,I). Finally, we investigated the role of CAFs in early and late recurrence of HCC. Compared with cases of late recurrence (over 2 years), HCC patients with early recurrence (less than 2 years) had higher levels of FAP expression (Figure 8G). These results suggest that S100A11 plays a crucial role in HCC development and progression and in regulating the TME. CAFs and M2 macrophages are key cell types that change within the tumor microenvironment, and S100A11 may promote the M2 transformation of macrophages by promoting CAFs activation.

### 3.9 IL-6 and peripheral blood CD $8^{+}$ T cells for predicting the response to immunotherapy in unresectable HCC

To further elucidate the role of the TME in immune resistance in HCC and identify noninvasive markers that can predict the efficacy of immunotherapy in HCC, we analyzed the differences in IL-6 and peripheral blood CD $8^{+}$ T cells between the immune-responsive and the immune-unresponsive groups. A total of 64 patients with unresectable HCC receiving ICIs were enrolled. The median age of the cohort was 61.5 years (range: 37–78 years), and 79.69% (51/64) were male. The primary etiology was hepatitis B (85.94%, 55/64). The Child–Pugh staging was grade A in 62.5% (40/64) and grade B in 37.5% (24/64). According to the Barcelona Clinic Liver Cancer (BCLC) staging, 64.06% (41/64) of the patients had advanced HCC. Among all the patients, 32.81% (21/64) received ICIs plus targeted therapy, and 67.19% (43/64) received ICIs plus targeted therapy and local therapy, including hepatic arterial infusion chemotherapy (HAIC) and trans-arterial chemoembolization (TACE). Among the 64 HCC patients, 42.19% (27/64) had a response rate assessment of PD, whereas 57.81% (37/64) had a response rate of PR or SD. Based on the initial response to immunotherapy, all patients were categorized into the immune-responsive group (PD, n = 27) and the immune-unresponsive group (PR/SD, n = 37). Table 1 shows no differences between the two groups in terms of age, sex, viral hepatitis, baseline AFP, PIVKA-II levels, Child–Pugh grade, or BCLC stage (Table 1). Table 2 shows significant differences in peripheral blood CD $8^{+}$ T cell counts and IL-6 between the two groups (Table 2). The IL-6 level in the immune-responsive group was significantly lower than that in the immune-unresponsive group (18.14 ± 14.40 pg/mL vs. 47.84 ± 45.78 pg/mL, *p* = 0.0038). The peripheral blood CD $8^{+}$ T cell count in the immune-responsive group was significantly higher than that in the immune-unresponsive group (0.32 ± 0.17* $10^{9}$ /L vs. 0.2 ± 0.11* $10^{9}$ /L, p = 0.001). Furthermore, studies have shown that PNI can predict survival in patients with advanced HCC treated with HAIC combined with targeted therapy and immunotherapy (Tang et al., 2025). Therefore, we compared the PNI between the two groups and found that the PNI in the immune-responsive group was significantly higher than that in the immune-unresponsive group (44.61 ± 6.07 vs. 40.39 ± 5.60, *p* = 0.006).

**TABLE 1:** Baseline characteristics between the patients in the responsive group and unresponsive group in immunotherapy.

Characteristics	Responsive group (n = 37)	Unresponsive group (n = 27)	*p*
Age ± SD (years old)	58.8 ± 9.4	61.9 ± 10.2	0.215
Gender	​	​	0.523
Male	31	20	​
Female	6	7	​
Virus hepatitis	​	​	0.065
HBV	31	24	​
HCV	5	3	​
Absent	1	0	​
AFP level (ng/mL)	​	​	0.424
< 400	20	11	​
≥ 400	17	16	​
PIVKA-II level (mAU/mL)	​	​	0.125
< 400	11	3	​
≥ 400	26	24	​
Child–Pugh grade	​	​	0.078
A	27	13	​
B	10	14	​
BCLC stage	​	​	0.245
B	16	7	​
C	21	20	​

**TABLE 2:** Hematological parameters between the patients in the responsive group and unresponsive group in immunotherapy.

Characteristics	Responsive group (n = 37)	Unresponsive group (n = 27)	*p*
PNI	44.61 ± 6.07	40.39 ± 5.6	0.006
CD $8^{+}$ T ( $10^{9}$ /L)	0.32 ± 0.17	0.2 ± 0.11	0.001
IL-6 pg/mL	18.14 ± 14.40	47.84 ± 45.78	0.004

**FIGURE 9:**
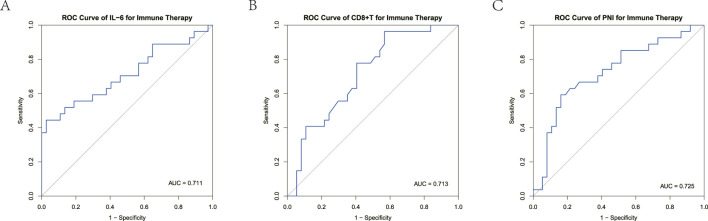
**(A)** ROC curves for the correlation of IL-6 levels with response to ICIs treatment in unresectable HCC patients. **(B)** ROC curves for the correlation of peripheral blood CD8+ T cells with response to ICIs treatment in unresectable HCC patients. **(C)** ROC curves for the correlation of PNI with response to ICIs treatment in unresectable HCC patients.

To determine whether IL-6, peripheral blood CD $8^{+}$ T cell count, and PNI can be used as predictors of immunotherapy response, we plotted ROC curves and calculated the AUC to evaluate the ability of IL-6, peripheral blood CD $8^{+}$ T cell count, and PNI to predict immunotherapy efficacy. The AUC for IL-6 was 0.711, with an optimal cutoff of 48.56 pg/mL (Figure 9A). The AUC for peripheral blood CD $8^{+}$ T cell count was 0.713, with an optimal cutoff of 0.35 * $10^{9}$ /L (Figure 9B). The AUC for PNI was 0.725, with an optimal cutoff of 40.08 (Figure 9C). These results suggest that IL-6, peripheral blood CD $8^{+}$ T cell count, and PNI have good predictive efficacy regarding the efficacy of immunotherapy for unresectable HCC patients. When IL-6 > 48.56 pg/mL, peripheral blood CD $8^{+}$ T cell count < 0.35 * $10^{9}$ /L, and PNI < 40.08, the efficacy of immunotherapy may be poor.

## 4 Discussion

Exosomes and their derived biomolecules have gained considerable attention due to their potential roles in the initiation and progression of various cancers, along with their functions as diagnostic biomarkers and therapeutic targets. Some exosome-derived molecules have been shown to be potential prognostic biomarkers for various cancers and can predict the efficacy of immunotherapy. Tao et al. (2022) developed a two-gene exosome-related gene signature (ADAMTS5 and G6PD) as a predictive model for HCC prognosis, which, as an independent predictor, could predict 1-, 2-, and 3-year survival rates. Du et al. (2024) developed a four-gene exosome-related gene signature (DYNC1H1, PRKDC, CCDC88A, and ADAMTS5) that could predict the prognosis of HCC patients and was significantly associated with the infiltration of various immune cells such as NK cells and Treg cells in the TME. In colorectal cancer, exosome-related gene signatures have also been developed for prognosis prediction. Cui et al. (2022) developed a three-gene signature (CCKBR, HOXC6, and HOXC6) that can not only predict the prognosis and chemotherapy sensitivity of CRC patients but also predict the efficacy of immunotherapy. The advent of immunotherapy has significantly improved the prognosis of HCC; however, approximately 50% of patients do not respond to initial immunotherapy, and nearly 30% of the responders subsequently develop secondary resistance (Qin et al., 2023). Therefore, early prediction of HCC patient prognosis and immunotherapy efficacy, elucidation of the mechanisms underlying immunotherapy resistance, and improvement of patient survival benefits are urgent issues that need to be addressed.

In this study, we used Cox regression and Lasso regression analyses to construct a prognostic signature integrating two prognostic TDEI genes (S100A11 and PUSL1) and combined it with clinical characteristics to construct a prediction model for HCC prognosis and immunotherapy efficacy. When the risk score is used alone to predict the prognosis of HCC patients, its predictive efficacy is moderate. When prediction OS is at 1, 2, and 3 years, the corresponding AUC values are less than 0.7. This could be due to an insufficient sample size (n = 371) limiting the model’s generalization ability or because the risk score is calculated from two feature genes, the limited number of which prevents it from fully reflecting the heterogeneity of HCC. When combined with clinico-pathological characteristics such as age and stage, the predictive model shows better predictive efficacy. When prediction OS is at 1, 2, 3, and 5 years, the corresponding AUC values of the ROC curev are all greater than or equal to 0.7. S100A11 expression levels were positively correlated with CAFs and M2 macrophage infiltration. S100A11 may promote CAF activation, which, in turn, promotes the polarization of macrophages toward the M2 phenotype, leading to an immunosuppressive TME and, ultimately, immune resistance. We also tested this hypothesis in clinical samples using IHC.

S100A11 is a S100 calcium-binding protein and a member of the S100 protein family (Zhang et al., 2024). It plays an important role in multiple biological processes, including inflammation, autoimmune diseases, and tumorigenesis. Regarding the promotion of liver fibrosis, studies have shown that S100A11 promoted liver fibrosis through the TGF- β -Smad2/Smad3 pathway (Zhu et al., 2022). In HCC, S100A11 can be used as a novel diagnostic biomarker in combination with AFP. In HCC, S100A11 regulates the proliferation, migration, invasion, and EMT of liver cancer cells through the AKT and ERK signaling pathways (Zheng et al., 2023). In terms of regulating the TME, liver cancer cell clusters with strong stemness characteristics and poor prognosis are closely associated with S100A11 gene expression levels and a large infiltration of M2 macrophages (Zhang et al., 2024). In addition to M2 macrophages, the infiltration of other immune cells in the TME can also be regulated by S100A11. Wang et al. found that in colorectal cancer, S100A11, as an exosome-related gene, was highly expressed in tumor tissue and regulated the infiltration and activity of immune cells such as neutrophils and Treg cells in the TME, leading to immune escape (Wang et al., 2025). The above studies confirm that S100A11 can alter the infiltration of lymphoid and myeloid immune cells and its role in reshaping the TME by altering the infiltration status of immune-activating and immunosuppressive cells. However, several studies have analyzed the role of exosome-derived S100A11 in HCC progression and immune resistance, particularly its role in regulating stromal cells in the TME.

Immune infiltration analysis and pathway enrichment analysis suggest possible mechanisms by which S100A11 regulates the TME. ESTIMATE and EPIC analyses have clarified the relationship between S100A11, stromal cells, and immune cells. High S100A11 expression increases the stromal score and CAF infiltration, and increased CAF infiltration increases M2 macrophage infiltration. CAFs are key pro-tumor components of the TME, contributing to tumor progression, angiogenesis, immune escape, and drug resistance (Affo et al., 2017). In prostate cancer, S100A11 leads to reduced CD $8^{+}$ T cell infiltration via CAFs (Han et al., 2024). In HCC, CAFs can induce the differentiation and aggregation of immunosuppressive cells such as N2 neutrophils and M2 macrophages by secreting cytokines, or they exert immunosuppressive functions by inhibiting cytotoxic cells such as T cells and NK cells (Gan et al., 2024; Yang et al., 2020; Chen S.-W. et al., 2021; Song et al., 2021). Promoting immunosuppressive cells and reducing cytotoxic cells are the basis for CAFs to regulate the TME. Our research revealed a positive correlation between M2 macrophages and S100A11. In addition, current research has shown that high expression of immunosuppressive molecules such as PD-L1, CTLA-4, and TIM-3 by M2 macrophages is a mechanism leading to immune escape and immune resistance (Yin et al., 2022; Chen J. et al., 2021; Palacios et al., 2022). In our research, we found that the expression of CTLA-4 and HAVCR2 were significantly elevated in the S100A11 high-expression group, whereas PD-L1 expression did not differ significantly between the two groups. Furthermore, GO analysis revealed that CAFs are associated with biological behaviors and pathways such as chemotaxis and cytokine–cytokine receptor interactions. Cytokines are key proteins in signal transduction in the TME and have multiple effects. Recruiting and regulating immune cells is one of their main functions (Kureshi and Dougan, 2025). This suggests that the relationship between S100A11 and the TME is likely to be the following: S100A11 promotes CAF activation, which, in turn, increases the expression of cytokines and chemokines by activated CAFs. These cytokines and chemokines promote the differentiation and migration of tumor-associated macrophages toward the M2 phenotype, leading to the expression of immunosuppressive molecules and, consequently, the formation of an immunosuppressive TME. Currently, there is little research on the recruitment and regulation of immunosuppressive cells by CAFs.

IL-6 is a multifunctional inflammatory cytokine with increased expression in various cancers, including HCC. Studies have shown that CAFs and TAMs are the main cells that secrete IL-6, and they can promote tumor progression by secreting IL-6 (Zhao et al., 2019; Cui et al., 2023). Furthermore, in HCC and non-small-cell lung cancer, IL-6 can predict the response to ICIs (Jeong et al., 2025; Miura et al., 2025). CD $8^{+}$ T cells are one of the main immune cells exerting antitumor effects and have been used to predict prognosis and drug resistance in HCC patients (Pu et al., 2022). Therefore, we evaluated the role of IL-6 and CD $8^{+}$ T cells in predicting the response to immunotherapy in unresectable HCC. We confirmed that higher IL-6 levels and lower peripheral blood CD $8^{+}$ T cell counts were associated with a poor response to immunotherapy, which is consistent with previous studies. The PNI, which represents the body’s nutritional and immune status, is increasingly recognized in HCC and has been used to predict the prognosis of liver transplantation or radical hepatectomy, along with the prognosis of combined targeted immunotherapy and HAIC (Tang et al., 2025; Kornberg et al., 2022; Fan et al., 2021). Our study found that PNI can also predict the response to HCC immunotherapy, with lower PNI indicating poor immunotherapy response.

Exosomes play a key role in regulating cytokine expression in CAFs, but no studies have yet combined exosome regulation of cytokine expression in CAFs with cytokine regulation of TAMs. Therefore, our study organically integrated these two components. KEGG analysis revealed that the expression of the MAPK signaling pathway, the PI3K-AKT signaling pathway, and the TGF- β signaling pathway, among others, increased when CAFs were highly infiltrated. These pathways have been implicated in the development and progression of HCC. Among them, the TGF- β signaling pathway is a key pathway for the transformation of fibrocytes into CAFs (Ringuette Goulet et al., 2018; Shi et al., 2020). This suggests that exosome S100A11 secreted by tumor cells is released into the TME and taken up by fibroblasts. S100A11 promotes the transformation of fibroblasts into CAFs through the TGF- β signaling pathway, which, in turn, promotes the transformation of macrophages into the M2 phenotype. M2 macrophages contribute to immune resistance by promoting the expression of immunosuppressive molecules. We detected S100A11, CAFs, and M2 at the protein level through IHC and qRT-PCR and demonstrated a positive correlation between the three, thus determining their value in predicting HCC prognosis. However, our experiments did not validate the TGF- β signaling pathway, nor did we investigate which cytokines released by CAFs are involved in the transformation of macrophages to the M2 phenotype. IL-6 may be the primary cytokine that contributes to immune resistance in CAFs. We will conduct subsequent cell-based experiments and expand the clinical sample size for peripheral blood analysis to address the limitations of this study. Regarding the predictive model, to improve its accuracy, future studies will include more samples and integrate other features such as radiomics features, exploring more advanced machine learning methods. Despite these limitations, our model still provides preliminary evidence for risk stratification of HCC patients and may lay the foundation for further model refinement.

## 5 Conclusion

In summary, we developed a prognostic model based on TDEI genes to predict the prognosis of HCC patients, changes in the TME, and the response to immunotherapy. This prognostic model can serve as an independent prognostic factor for HCC patients and highlights the function and significance of TDEI genes. In addition, IL-6, peripheral blood CD $8^{+}$ T cell counts, and PNI can be used as predictors of the response to immunotherapy in patients with unresectable HCC.

## Data Availability

The TCGA-LIHC, ICGC-LIRI-JP, and GSE181946 datasets analyzed in this study are publicly available. Original experimental data and all other data supporting the conclusions of this article are included within the article and its supplementary material or are available from the corresponding author upon reasonable request.
