# Supplementary material for: Exosome-related immune signatures and peripheral blood assays predict prognosis and immunotherapy response in hepatocellular carcinoma
Source: Front Cell Dev Biol. 2026 Jan 26;13:1696790. doi: 10.3389/fcell.2025.1696790 (PMC12883759; doi:10.3389/fcell.2025.1696790)
Supplement: Supplementary file 1 [file DataSheet2.pdf]

Supplementary Material

1 SUPPLEMENTARY FIGURE

1.1 Figures

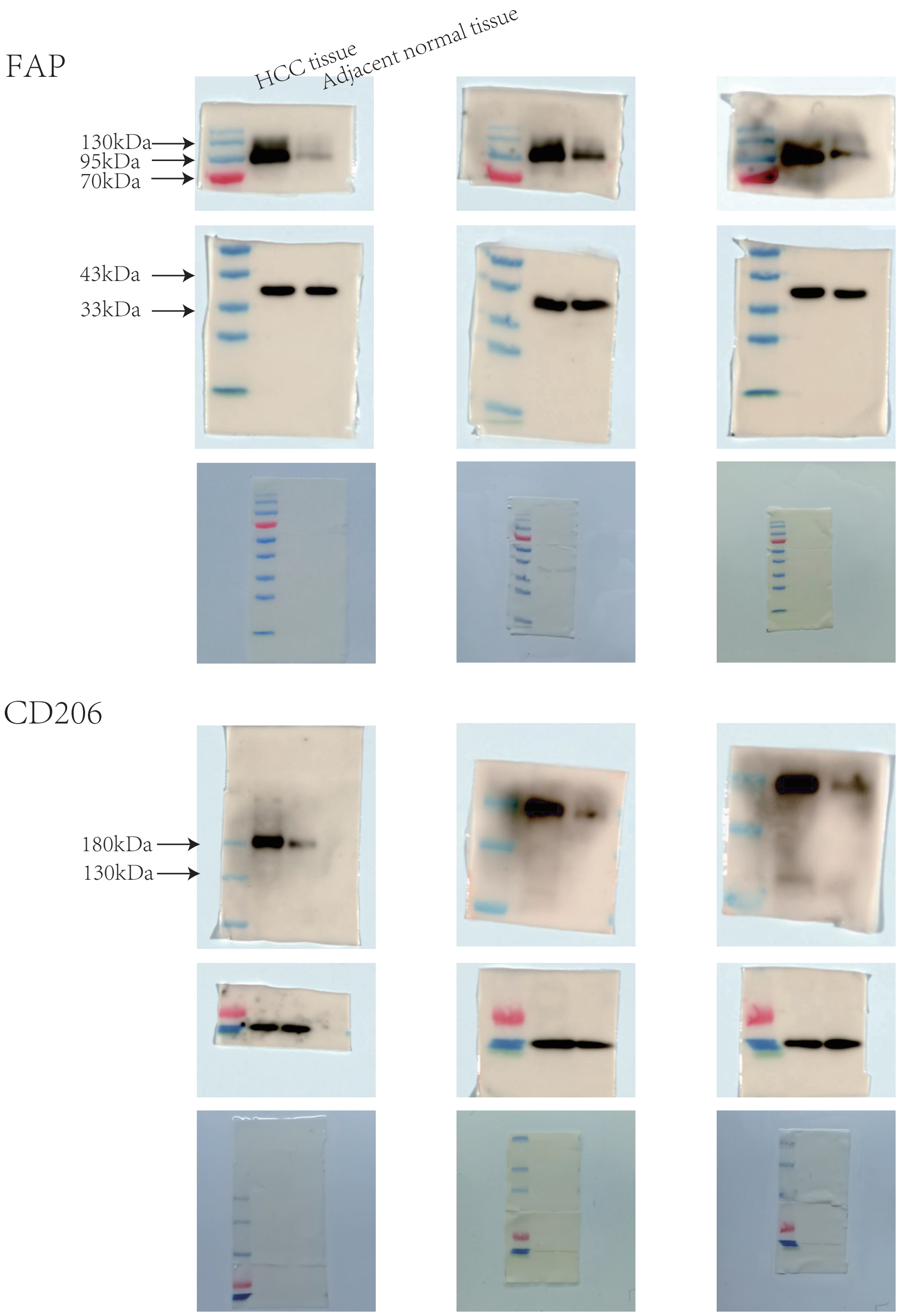

Figure S1. Results of Western Blot.

1.2 Figures

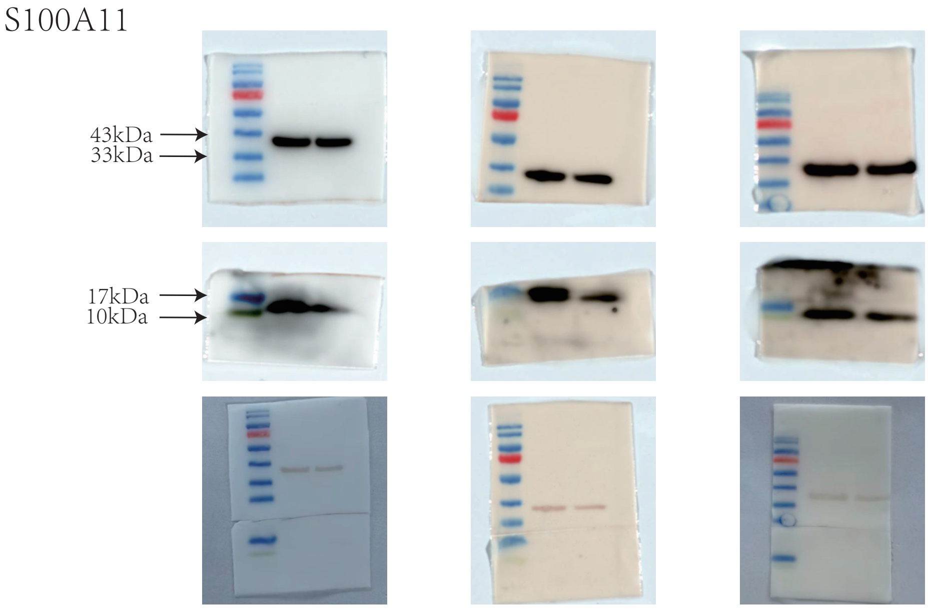

Figure S2. Results of Western Blot.
