# Supplementary material for: Exosome-related immune signatures and peripheral blood assays predict prognosis and immunotherapy response in hepatocellular carcinoma
Source: Front Cell Dev Biol. 2026 Jan 26;13:1696790. doi: 10.3389/fcell.2025.1696790 (PMC12883759; doi:10.3389/fcell.2025.1696790)
Supplement: Supplementary file 2 [file DataSheet1.pdf]

Supplementary Material

1 SUPPLEMENTARY TABLE

Genes	Sequences
S100A11	Forward: 5'-CGAGTCCCTGATTGCTGTCT-3'
	Reverse: 5'-TCATCATGCGGTCAAGGACA-3'
FAP	Forward: 5'-TCGATACCACTTACCCTGCG-3'
	Reverse: 5'-AGTAACCCACGTGAGCCAAC-3'
CD206	Forward: 5'-GTGTTGTTATTATTGGAGGTGCAT-3'
	Reverse: 5'-GCTGGAGGATTAGTCAAGGAA-3'
β-actin	Forward: 5'-ACTTAGTTGCGTTACACCCTT-3'
	Reverse: 5'-GTCACCTTCACCGTTCCA -3'

Table S1. Primer sequences for qPCR
